# Implementing antenatal care recommendations, South Africa

**DOI:** 10.2471/BLT.20.278945

**Published:** 2021-01-21

**Authors:** Tsakane MAG Hlongwane, Burcu Bozkurt, Maria C Barreix, Robert Pattinson, Metin Gülmezoglu, Valerie Vannevel, Özge Tunçalp

**Affiliations:** a South African Medical Research Council/University of Pretoria Maternal and Infant Health Care Strategies Unit, Department of Obstetrics and Gynaecology, University of Pretoria, Unit Private Bag X323 Arcadia, Pretoria 0007, South Africa.; b Gillings School of Global Public Health, The University of North Carolina at Chapel Hill, Chapel Hill, United States of America.; c Department of Sexual and Reproductive Health and Research, World Health Organization, Geneva, Switzerland.

## Abstract

Despite progress in reduction in maternal deaths in South Africa, deaths due to complications of hypertension in pregnancy remain high at 26 deaths per 100 000 live births in 2016. The South African health ministry modified its existing four-visit antenatal care model to align with the World Health Organization’s (WHO) 2016 recommendations for the number and content of antenatal care contacts. Implementation of the eight-contact antenatal care recommendations began in April 2017, after adaptation to the national context and nationwide trainings. In this article, we describe the stages of implementation and the monitoring of key indicators. We share lessons, particularly from the important early stages of nationwide scale-up and an analysis of the early results. We analysed samples of maternity case records in four catchment areas in the first year of the updated care model. The mean number of antenatal care contacts among five monthly samples of 200 women increased steadily from 4.76 (standard deviation, SD: 2.0) in March 2017 to 5.90 (SD: 2.3) in February 2018. The proportion of women with hypertension detected who received appropriate action (provision of medical treatment or referral) also increased from 83.3% (20/24) to 100.0% (35/35) over the same period. South Africa’s experiences with implementation of the updated antenatal care package shows that commitment from all stakeholders is essential for success. Training and readiness are key to identifying and managing women with complications and developing an efficient antenatal care system accessible to all women.

## Résumé

En dépit des progrès observés dans la diminution des décès maternels en Afrique du Sud, les complications causées par l'hypertension durant la grossesse entraînent encore des pertes élevées, à savoir 26 sur 100 000 naissances en 2016. Le ministère de la Santé sud-africain a modifié son modèle de soins prénatals en quatre visites afin de s'aligner sur les recommandations 2016 de l'Organisation mondiale de la Santé (OMS) relatives au nombre et au type de contacts de soins prénatals. La mise en œuvre des recommandations de soins prénatals prévoyant au moins huit contacts a débuté en avril 2017, après adaptation au contexte du pays et formation à l'échelle nationale. Dans cet article, nous décrivons chaque étape de cette mise en œuvre et la surveillance des principaux indicateurs. Nous partageons les enseignements tirés, en particulier lors des premières étapes cruciales d'intensification au niveau national et d'analyse des résultats initiaux. Nous avons étudié des échantillons de dossiers de maternité dans quatre circonscriptions au cours de la première année d'instauration du nouveau modèle de soins. Le nombre moyen de contacts de soins prénatals sur cinq échantillons mensuels de 200 femmes a augmenté de manière stable, passant de 4,76 (écart type: 2,0) en mars 2017 à 5,90 (écart type: 2,3) en février 2018. Le pourcentage de femmes chez qui une hypertension a été détectée et qui ont bénéficié d'une prise en charge adéquate (traitement médical ou renvoi vers un spécialiste) a également augmenté, de 83,3% (20/24) à 100,0% (35/35) sur la même période. L'expérience de l'Afrique du Sud dans la mise en œuvre du nouveau modèle de soins prénatals montre qu'un engagement de la part de tous les intervenants est essentiel à la réussite de l'initiative. La formation et la préparation sont indispensables pour identifier et prendre en charge les femmes présentant des complications, mais aussi pour développer un système de soins prénatals efficace et accessible à toutes.

## Resumen

A pesar de los progresos realizados en cuanto a la reducción de las muertes maternas en Sudáfrica, las muertes por complicaciones debido a la hipertensión durante el embarazo siguen siendo elevadas, con 26 muertes por cada 100.000 nacidos vivos en 2016. El Ministerio de Salud de Sudáfrica modificó su actual modelo de atención prenatal de cuatro visitas para ajustarlo a las recomendaciones de la Organización Mundial de la Salud (OMS) para 2016 en cuanto al número y el contenido de los contactos de atención prenatal. La aplicación de las recomendaciones de atención prenatal de ocho visitas comenzó en abril de 2017, tras su adaptación al contexto nacional y la formación a nivel nacional. En el presente artículo se describen las etapas de aplicación y el seguimiento de los indicadores clave. Compartimos las lecciones, en particular, las importantes etapas iniciales de la ampliación a escala nacional y un análisis de los primeros resultados. Analizamos muestras de registros de casos de maternidad en cuatro zonas de captación en el primer año del modelo de atención actualizado. El número medio de contactos de atención prenatal entre cinco muestras mensuales de 200 mujeres aumentó de forma constante de 4,76 (desviación estándar, SD: 2,0) en marzo de 2017 a 5,90 (SD: 2,3) en febrero de 2018. La proporción de mujeres con hipertensión detectada que recibieron medidas adecuadas (suministro de tratamiento médico o remisión) también aumentó del 83,3% (20/24) al 100,0% (35/35) en el mismo período. Las experiencias en Sudáfrica en cuanto a la aplicación del conjunto de medidas actualizadas de atención prenatal demuestran que el compromiso de todos los interesados es esencial para el éxito. La formación y la preparación son fundamentales para identificar y tratar a las mujeres con complicaciones, así como para desarrollar un sistema eficiente de atención prenatal accesible a todas las mujeres.

## ملخص

على الرغم من التقدم الذي تم تحقيقه في الحد من وفيات الأمهات في جنوب إفريقيا، فإن الوفيات الناجمة عن مضاعفات ارتفاع ضغط الدم أثناء الحمل تظل مرتفعة بمعدل 26 حالة وفاة لكل 100000 حالة ولادة حية في عام 2016. قامت وزارة الصحة في جنوب إفريقيا بتعديل نموذجها الحالي للرعاية خلال فترة الحمل والمكون من أربع زيارات، ليتماشى مع توصيات منظمة الصحة العالمية (WHO) لعام 2016 بشأن عدد ومحتوى جهات اتصال الرعاية خلال فترة الحمل. بدأ تنفيذ توصيات الرعاية خلال فترة الحمل، والمرتكزة على ثمان جهات اتصال، في أبريل/نيسان 2017، بعد التكيف مع السياق الوطني والدورات التدريبية على الصعيد الوطني. نقوم في هذا المقال بوصف مراحل التنفيذ ورصد المؤشرات الرئيسية. نحن نقوم بمشاركة الدروس، وخاصة من المراحل المبكرة الهامة من التوسع الوطني وتحليل النتائج المبكرة. قمنا بتحليل عينات من سجلات حالات الأمهات في أربع مناطق للتجمعات في السنة الأولى من نموذج الرعاية المحدث. زاد متوسط ​​عدد جهات الاتصال للرعاية خلال فترة الحمل بين خمس عينات شهرية من 200 امرأة بثبات من 4.76 (الانحراف المعياري، SD: 2.0) في مارس/آذار 2017 إلى 5.90 (الانحراف المعياري: 2.3) في فبراير/شباط 2018. نسبة النساء اللاتي تم اكتشاف إصابتهن بارتفاع ضغط الدم، واللاتي حصلن على الإجراء المناسب (توفير العلاج الطبي أو الإحالة)، زاد أيضًا من 83.3% (20/24) إلى 100.0% (35/35) خلال نفس الفترة. إن تجارب جنوب إفريقيا في تنفيذ حزمة الرعاية المحدثة أثناء فترة الحمل، توضح أن الالتزام من جميع الأطراف المعنية ضروري لتحقيق النجاح. يعد كل من التدريب والاستعداد مفتاحًا لتحديد النساء اللواتي يعانين من مضاعفات، فضلاً عن التعامل معهم، وكذلك تطوير نظام فعال للرعاية أثناء فترة الحمل، وجعله في متناول جميع النساء.

## 摘要

尽管南非在减少孕产妇死亡方面取得了进展，但由于妊娠高血压并发症导致的死亡人数仍然很高，2016 年每 100 000 例活产中有 26 人死亡。南非卫生部修改了其现有的四次产前检查模式，以符合世界卫生组织 (WHO) 2016 年有关产前保健接触次数和内容的建议。适应国情并在全国范围内进行培训后，于 2017 年 4 月开始实施八次接触的产前保健建议。在本文中，我们说明了实施阶段和关键指标的监测情况。我们特地分享了在全国范围内重要的纵向拓展早期阶段总结的经验教训以及对早期结果进行的分析。我们分析了在更新保健模式的第一年中四个流域的产妇病例记录样本。在五个月 200 名女性的样本中，产前保健接触的平均次数从 2017 年 3 月的 4.76（标准差，SD：2.0）稳步上升至 2018 年 2 月的 5.90（SD：2.3）。我们发现在同一时期接受适当措施（接受医护治疗或转诊）的高血压女性患者的比例也从 83.3% (20/24) 增加至 100.0% (35/35)。南非在实施更新的产前保健一揽子计划方面的经验表明，所有利益相关者的贡献对于成功至关重要。培训和准备情况是鉴定与诊治有并发症的女性以及开发一个所有女性均可享用的有效产前保健系统的关键。

## Резюме

Несмотря на прогресс в сокращении материнской смертности в Южной Африке, смертность от осложнений по причине гипертензии во время беременности остается высокой и составляет 26 случаев на 100 000 живорожденных в 2016 году. Министерство здравоохранения Южной Африки изменило существующую модель оказания дородовой помощи с четырьмя посещениями, чтобы привести ее в соответствие с рекомендациями Всемирной организации здравоохранения (ВОЗ) от 2016 года в отношении количества и содержания контактов с учреждением дородовой помощи. Реализация рекомендаций о восьми контактах с учреждением по дородовой помощи началась в апреле 2017 года после адаптации к национальному контексту и общенациональных тренингов. В этой статье описаны этапы реализации и мониторинга ключевых показателей. Авторы делятся опытом, в частности полученным на важных ранних этапах расширения до общенационального масштаба, и опытом анализа ранних результатов. Диспансерные книжки беременных были выборочно проанализированы в четырех зонах обслуживания в первый год применения обновленной модели оказания помощи. Среднее количество контактов с учреждением дородовой помощи среди пяти ежемесячных выборок из 200 женщин стабильно увеличивалось с 4,76 (стандартное отклонение, СО: 2,0) в марте 2017 года до 5,90 (СО: 2,3) в феврале 2018 года. Доля женщин с выявленной артериальной гипертензией, в отношении которых были приняты надлежащие меры (лечение или направление к специалисту), также увеличилась с 83,3% (20/24) до 100,0% (35/35) за тот же период. Опыт Южной Африки по реализации обновленного пакета услуг по дородовой помощи показывает, что приверженность делу всех заинтересованных сторон имеет важное значение для успеха. Обучение и готовность — это ключ к выявлению и ведению женщин с осложнениями, а также к разработке эффективной системы дородовой помощи, доступной для всех женщин.

## Introduction

South Africa is a middle-income country with a population of more than 59 million.^1^ In 2017, sub-Saharan Africa had the highest estimated maternal mortality ratio worldwide at 542 deaths per 100 000 live births and stillbirths at 28.7 per 1000 total births.^2^ While progress has been made in sub-Saharan Africa, the sustainable development goals and the United Nations’ *Global strategy for women’s, children’s and adolescents’ health (2016–2030)* seek to improve on gains and aim to reduce the global maternal mortality ratio to 70 deaths per 100 000 live births.^3^^,^^4^ Access to quality antenatal care is crucial to achieve these goals and improve the lives of both mothers and babies.^5^^,^^6^ Antenatal care is also a key component of the continuum of care for women, babies and their families. While most women in low- and middle-income countries attend at least one antenatal care visit, the content and quality of these contacts are not standardized.^7^ Therefore, it is necessary to improve antenatal care services and to update indicators that track antenatal care performance.

Evidence of a higher risk of perinatal death associated with four or fewer antenatal care visits prompted the World Health Organization (WHO) to develop a new guideline.^8^ In November 2016, WHO released its comprehensive *Recommendations on antenatal care for a positive pregnancy experience*,^9^ prioritizing person-centred care and well-being as well as prevention of mortality and morbidity.^10^ Following these recommendations, in April 2017 the South African health ministry modified its existing four-visit basic antenatal care model to align with the new WHO recommendations including the number of contacts, content of each contact (such as respectful care and clinical enquiry for intimate partner violence) and the basic skills needed for antenatal care.

The nationwide scale-up of South Africa’s updated package of care, called Basic Antenatal Care Plus, aimed to improve the quality of antenatal care through the provision of evidence-based interventions across a minimum of eight antenatal care contacts, with an increased number of contacts during the third trimester. The package aims to improve pregnancy care, outcomes and women’s experiences, yielding an improvement in screening and detection of pregnancy-related complications and, ultimately, to improve antenatal care quality.^11^ In this paper we document the adaptation and implementation of the WHO 2016 antenatal care recommendations in South Africa and share some lessons learnt, particularly in the crucial early stages of nationwide scale-up.

## Local setting

There are 52 districts in South Africa, with a majority having more than 500 000 inhabitants.^1^ Since 2008 South Africa has been implementing a basic antenatal care model to achieve four goal-orientated visits. An audit of the timing and causes of all stillbirths in three of South Africa’s provinces (Limpopo, Mpumalanga and Western Cape) from 2013 to 2015 showed that hypertensive disorders of pregnancy and unexplained stillbirths were the most common adverse outcomes.^12^ The third trimester was identified as a crucial time, with a peak in stillbirths between 32 and 38 weeks. While the majority (75.5%) of pregnant women in South Africa attended four antenatal care visits in 2016,^13^ due to the extended period in the schedule between 32 and 38 weeks, stillbirths were diagnosed but not being prevented. Additionally, although maternal deaths due to non-pregnancy-related infections fell by 47% (from 59 deaths per 100 000 live births in 2011 to 31 deaths per 100 000 in 2016), deaths due to complications of hypertension in pregnancy remained high (14% increase from 23 deaths per 100 000 live births to 26 deaths per 100 000 during the same period).^14^ Increased antenatal care contacts in the third trimester may help to identify and prevent hypertension-related complications.^12^^,^^15^

## Public health approach

The updated antenatal care recommendations were adapted and implemented into clinical practice following a systematic approach along four steps: (i) developing the guidelines, related protocols and training materials; (ii) preparing the necessary resources and training; (iii) implementing the updated care package across the country; and (iv) monitoring and evaluation.

### Developing guidelines and training 

Two ministerial committees – the South African National Committee for the Confidential Enquiries into Maternal Deaths and the National Perinatal Morbidity and Mortality Committee – and a health ministry task team reviewed the WHO 2016 antenatal care recommendations. The committees also reviewed local data which demonstrated the association between the number of antenatal care visits (fewer than eight contacts) and increased maternal and perinatal mortality and morbidity. The task team presented a report detailing the implications and findings to the ministerial teams. Following discussions, the South African Medical Research Council Maternal and Infant Health Strategies Unit and major stakeholders (subject matter experts, academics and professional bodies) updated the basic antenatal care guidelines and prepared a training package for staff. 

The training package included: guidance for heads of departments and facility managers on organizing antenatal care; a guide for training facilitators; a guide for trainers; health-care facility manager’s notes and referral hospital notes; checklists; a task book; an antenatal care handbook; protocol guides; and standards of operation for all health-care professionals offering antenatal care to facilitate the incorporation of the changes. For example, the checklists and related documents detailed the recommended content for each antenatal care contact with a pregnant woman, including the rationale behind the interventions and actions. The package contained guidance on how to identify, triage, investigate, manage and arrange follow-up and referral of all pregnant women. Government ministerial committees and the Medical Research Council’s Maternal and Infant Health Care Strategies Unit played an integral role in adapting the 2016 WHO recommendations to the South African clinical context. It was important to ensure that all the steps for implementing change were taken based on the theory of change used by the research council for their maternal and infant programmes.^16^

The updated model of antenatal care was presented at a meeting of the South African health ministry (National Health Council) in November 2016 and accepted by the ministry, who then instructed the members of the executive council to implement the updated recommendations beginning 1 April 2017.

### Mobilizing resources and training 

The health ministry commissioned the Medical Research Council’s Maternal and Infant Health Care Strategies Unit to prepare the implementation package and to facilitate workshops to ensure every health-care worker providing antenatal care was familiar with the updated package of care. The implementing stakeholders (Medical Research Council, health ministry, ministerial committees and heads of provincial and district departments) facilitated stakeholders’ meetings, compiled the relevant training materials, conducted the training-of-trainers for district clinical specialist teams, and trained health-care providers. The initial training was held in all provinces (usually in a centralized location) and consisted of training-of-trainers for a range of stakeholders including managers, administrators and health-care workers from different levels. The role of the district clinical specialist teams was to ensure quality in clinical services; to provide clinical mentorship, monitoring and evaluation; and to support the health system and district-level organizational activities and clinical training. These teams therefore played a central role in connecting all facilities within the districts. The district teams used different methods depending on the unique circumstances of their catchment area. Training activities included: learning the new antenatal care contact schedule; using checklists, protocol books and standard operational manager’s booklets; and ensuring referral to the appropriate level of care for women with complicated conditions. Overall, there were six key stakeholder discussions and eight provincial training-of-trainers workshops where 334 provincial and district teams were trained.

The implementation of the updated care package was supported via additional resources (handbooks, checklists), training by specialized trainers and the district clinical specialist teams, and planned supervision visits from the district clinical specialist teams and the maternal, child and women’s health coordinators. The training packages were distributed through the provincial and district maternal, newborn and child health offices and to all Essential Steps in Managing Obstetric Emergencies trainers. Essential Steps in Managing Obstetric Emergencies is a focused training programme to support and build the capacity and confidence of health-care workers to enable them to work effectively and efficiently to deliver emergency obstetric care to reduce maternal and neonatal mortality in South Africa.^17^^,^^18^ Trainers, provincial teams, district clinical specialist teams, managers and health-care providers supported the roll-out of training across the country. Health-care providers were given a one-day training on the updated care package at a central location or at health facilities.

Staff in primary health-care clinics, community health centres and hospitals continued to provide the services required for the new model of antenatal care. Managers planned with their teams for changes in how to triage and to ensure that pregnant women received the appropriate care as described in the updated recommendations.

### Guideline implementation 

#### Dissemination and implementation

The health ministry was responsible for disseminating information on the updated antenatal care package to over 3000 public health-care facilities throughout South Africa. Plans for initial and continued scale-up aimed to facilitate and guide implementation across the country. The stakeholders engaged in this implementation process and their roles are summarized in Table 1. The government also disseminated information on the benefits of the new model of care to the public through educational leaflets, national radio stations and other media platforms. Examples of innovative dissemination include MomConnect, an initiative to support maternal health using cell phone-based technologies integrated into maternal and child health services;^19^ and Phila, an online government platform to provide health information and encourage healthy living.^20^ Messages on these platforms, and others, focused on the increase from four to eight antenatal care contacts, on the benefits of the eight-contact care model and its related schedule, and on general antenatal care tips.

**Table 1:** Stakeholders’ roles and tasks in the implementation of the World Health Organization’s 2016 antenatal care recommendations, South Africa

Role or task	Stakeholder				
	National Committee for Confidential Enquiry into Maternal Deaths and National Perinatal Morbidity and Mortality Committee	Health ministry	South African Medical Research Council, Maternal and Infant Health Care Strategies Unit	District clinical specialist teams, maternal, child and women’s health coordinators and heads of departments^a^	Emergency medical services
Role	Address high maternal mortality ratio by improving recording and analysis of maternal deaths	Approve and lead implementation process of Basic Antenatal Care Plus programme	Coordinate implementation of new guideline; Facilitate and organize stakeholders and districts	Provide clinical mentorship and guidance to health facilities to improve their ability to provide effective maternal, neonatal and child health services	Provide emergency transport services for all patients
Task	Audit reviews and compile Saving Mothers, Saving Babies report; Report findings to health ministry	Make decisions regarding new guideline and its implementation; Appoint stakeholders to implement updated guideline; Approve new guideline, protocols and permission for trainings	Outline WHO’s 2016 recommendations on antenatal care for a positive pregnancy experience; Update existing national maternal health guideline; Update existing antenatal care to Basic Antenatal Care Plus programme to align with WHO’s 2016 antenatal care recommendations; Coordinate teams: stakeholders and districts; Facilitate training-of-trainers workshops for provincial teams to implement Basic Antenatal Care Plus	Be trained and knowledgeable about the new guideline; Conduct trainings across districts and facilities; Monitor implementation process at district level	Provide inter-facility transfers for mothers and babies; Provide home-to-health facility transport for mothers and babies

^a^ Including obstetrics and gynaecology and nursing departments.

#### Supporting sustainability

Adoption of the updated recommendations into clinical practice was enhanced through the managers and district teams working together. District clinical specialist teams have continued to support and mentor facilities in their districts. Trainers have continued training, with one-day refresher sessions conducted within their facilities. Facility managers follow standards of operations and ensure that there is continuous learning and reinforcement of the principles of the care model. This process was enhanced by incorporating an antenatal care training module into the three-day Essential Steps in Managing Obstetric Emergencies training. Every district has individuals trained in the Essential Steps method. Health-care facilities across the country have trained individuals in Essential Steps and receive continuous professional development points for every module of the programme that they perform during the year.

Effective communication was essential for implementation and sustainability. Managers at all levels of care and individual health-care providers and emergency personnel were informed of the new guidelines through provincial and district teams including district clinical specialists; maternal, child and women’s health coordinators; and staff from academia and professional bodies. Health-care managers shared the information with their clinical and support staff. Provincial and district reporting lines ensured continuous communication and feedback of information from the health-care providers and managers at the various levels of care. Additionally, continuous trainings and refresher sessions for health-care providers to reinforce the guideline allowed for real-time monitoring of progress in implementation and for provincial and district teams to proactively address logistical challenges.

### Monitoring and evaluation

#### Before implementation

Before implementation of the updated care package, two-day focus groups were held with key stakeholders in districts around the country in January to March 2017 to discuss the implementation (Box 1). Important challenges identified included: increased workload due to the increased antenatal care contacts and the possible increase in referrals of women with high-risk pregnancies; lack of availability of transport to clinics for women with high-risk pregnancies; and possible shortage of hospital beds for admitting referrals.

Box 1 **Identifying challenges to implementation of the World Health Organization’s 2016 antenatal care recommendations in South Africa**

Before implementation of the updated antenatal care package, two-day workshops on antenatal care and emergency medical services were held in January to March 2017. During the workshops the Maternal and Infant Health Care Strategies Unit of the South African Medical Research Council facilitated a series of focus groups in six of the nine provinces (Mpumalanga, North West, Gauteng, Eastern Cape, Free State and Northern Cape) which comprise 31 of the 52 districts in the country. A total of 22 focus groups were held with 329 participants. Participants included all cadres from the health department, provincial, districts and facility representatives within the maternal and child health programme and emergency medical unit. The focus groups described the maternal, stillbirth and neonatal mortality for the province, district and facilities. This presentation was followed by discussions of antenatal care cases and led to brainstorming of innovative solutions to overcome implementation issues.

Multidisciplinary implementation teams in the focus groups identified four approaches to manage the likely increased load of high-risk pregnancies. First, an advanced antenatal care practitioner (advanced midwife or doctor) would be trained to see the referrals at primary health-care clinics once a week. These practitioners would decide whether to manage the woman at the clinic or refer her to the next level of care, such as a hospital. The practitioners would also have a direct contact to the hospital and specialists. Support would be done on a rotational basis allowing one professional nurse or doctor with specific training to service several clinics. The second approach was to train the doctors of the primary care clinics to see referrals. The doctor would decide whether to manage the woman at the clinic or refer her to the hospital or specialist. The Maternal and Infant Health Care Strategies Unit developed a training course for this purpose. A third approach was outreach visits by doctors from the hospital maternity unit to see women at the primary care clinic, mostly at midwife obstetric care units. A fourth approach was outreach by district clinical specialist teams to see women at the primary care clinics. Advanced midwives or family physicians from the district clinical specialist teams would be suitable for this role. 

Solutions for women who could not provide their own transport included planned patient transport run by the emergency medical units or use of maternity waiting homes for some women with high-risk pregnancies. 

Data collected showed that between 1 February 2017 and 1 April 2017, 524 health-care personnel from 46 districts had been trained on the updated care package before implementation at scale was initiated.

#### During implementation

Facility or district clinical specialists led audits of case records using a monitoring and evaluation system based on audit tools and a patient checklist. The aim was to ensure that guideline actions were being implemented as intended and that potential problem areas were being identified and adequately documented. 

We documented the numbers of antenatal care contacts and the detection and management of hypertension in samples of women over the first year (March 2017 to February 2018; Box 2). We extracted data retrospectively from 50 consecutive patient charts of women who gave birth in four geographically diverse catchments areas in five selected months (200 women in each phase, a total of 1000 women). Most of the women were between 24 and 26 years old. These results show an increase in total antenatal care contacts and hypertension detection. The mean total number of antenatal care contacts increased from 4.76 (standard deviation, SD: 2.0) in March 2017 to 5.9 (SD: 2.3) in February 2018 (Table 2). Almost all the women in each sample phase had their blood pressure measured at every contact, while detection of hypertension showed an overall increasing trend over time. The proportion of women with hypertension detected who received appropriate action (provision of medical treatment or referral) increased from 83.3% (20/24) in March 2017 to 100.0% (35/35) in February 2018.

Box 2 **Monitoring initial implementation of the World Health Organization’s 2016 antenatal care recommendations in South Africa**

We documented the numbers of antenatal care contacts and success in detection and management of hypertension over the first year after implementation of the updated antenatal care package.

We included four geographically diverse catchment areas based on the following criteria: more than 2500 deliveries per annum and the commensurate antenatal care attendance; established referral routes; and familiarity with the perinatal problem identification programme. The areas encompassed urban, semi-urban and rural areas to represent the different landscapes across South Africa: Ekurhuleni (two clinics and one hospital) in Gauteng province; Mafikeng (two clinics and one hospital) in North West province; Thohoyandou (one health centre and one hospital) in Limpopo province; and Upington (four clinics and one hospital) in Northern Cape province. The audited catchment areas across four districts represented 5.9 million inhabitants in those districts.

We extracted data retrospectively from 50 consecutive patient charts of women who gave birth in March, July, September, November 2017 and February 2018 in the four areas (a total of 1000 women). We recorded the total number of antenatal care contacts in each month of data collection and calculated the mean (and standard deviation) number of contacts per woman. We calculated the proportion of women having their blood pressure measured at each contact, the proportion with hypertension detected and the proportion of women receiving appropriate action when elevated blood pressure was identified. Ethical approval for data collection was obtained from the ethics committee of the University of Pretoria (approval no. REC 473/2014).

**Table 2:** Monitoring initial implementation of the World Health Organization’s 2016 antenatal care recommendations in selected sites, South Africa, March 2017 to February 2018

Month and year	Total no. of women sampled	Mean (SD) no. of antenatal care contacts per woman		No. (%) of women		
				Having blood pressure measured at each contact	With hypertension detected	Receiving appropriate action when elevated blood pressure identified^a^
March 2017	200	4.76 (2.0)		185 (92.5)	24 (12.0)	20 (83.3)
July 2017	200	4.87 (2.2)		185 (92.5)	27 (13.5)	24 (88.9)
September 2017	200	5.46 (2.2)		197 (98.5)	23 (11.5)	21 (91.3)
November 2017	200	5.36 (2.4)		190 (95.0)	39 (19.5)	36 (92.3)
February 2018	200	5.90 (2.3)		197 (98.5)	35 (17.5)	35 (100.0)

SD: standard deviation.

^a^ The denominator is the number of women who had hypertension detected.

Note: Implementation of the updated antenatal care package began on 1 April 2017. We analysed 50 consecutive antenatal care contacts in four areas in each month, a total of 1000 women (Box 2).

### Evaluating health outcomes

The effect of the updated package of care on maternal and perinatal outcomes nationally has not yet been rigorously evaluated. However, the observed trends in maternal and perinatal mortality since implementation of the package are encouraging. Evidence from 2019 demonstrates that South Africa has been able to reduce its maternal mortality for the first time in over a decade (from 189 deaths per 100 000 live births in 2009 to 99 deaths per 100 000 live births in 2019), with a reduction in almost all underlying causes of maternal mortality.^21^ In South Africa, where hypertensive disorders of pregnancy are the most common direct cause of maternal mortality and account for 14.8% of all maternal deaths, efforts to manage hypertensive disorders of pregnancy are important for detecting potentially preventable maternal deaths.^22^ Maternal deaths in health facilities due to hypertensive disorders of pregnancy were 23, 20 and 19 per 100 000 live births in 2017, 2018 and 2019, respectively, compared with an overall institutional maternal mortality ratio of 126, 118 and 99 per 100 000 live births, respectively.^21^ A trend of decreasing perinatal mortality has been shown in Mpumalanga district.^23^ Furthermore, data from 2017 identified major health-system factors associated with poor care of women with hypertensive disorders of pregnancy, which the updated care package aims to alleviate.^24^^,^^25^ Health-system problems identified included a lack of proper assessment and making a diagnosis at the primary level of care. Moreover, not adhering to standard protocols was the most significant problem at regional, tertiary and national central hospitals.^24^

## Lessons learnt and challenges

Our experience in South Africa shows that with commitment and allocation of resources, evidence-based recommendations can be implemented at scale. For implementation and adaptation, it was important that the health ministry led in committing to change and improving outcomes for pregnant women and their children. This commitment was necessary as districts relied on the ministry’s approved guidelines in their facilities. Following the ministry’s lead, other relevant stakeholders in all provincial departments, academic faculties, professional bodies and health-care provider forums were engaged to drive the changes and to commit to supporting all the steps of pre-implementation, implementation and monitoring and evaluation.

Implementation of the eight-contact antenatal care model posed new challenges to the South African health system, which other countries can learn from (Box 3). While antenatal care contacts are generally increasing, these gains are uneven. In our analysis, 23 (2.3%) of the sample of 1000 women across our four areas of study had zero contacts before birth since implementation of the updated care package. We also noted regional differences between the catchment areas (urban, semi-urban and rural communities). Factors such as population size, health-seeking behaviour, resources and staff capacity may exacerbate heterogeneity in implementation.^26^ Thus, implementation and scale-up plans must respond to the unique circumstances and challenges that each district may face across the continuum of the health system. An unintended consequence of the new care model could be an initial decrease in quality of care, as more antenatal care contacts could mean less time per woman. The workload at both primary health-care clinics and referral hospitals has increased during this period. To offset this, the maternal, child and women’s health coordinator, district clinical specialist teams and training-of-trainers staff visited primary health-care clinics, health centres and hospitals to support and train health-care providers as part of the implementation. Some facilities employed a booking system for routine antenatal care.

Box 3 **Summary of main lessons learnt**

• Commitment from all stakeholders and main decision-makers is necessary for successful implementation of the new antenatal care package at scale.

• Preparation and training are key to identifying women with high-risk pregnancies and referral to the appropriate services.

• Monitoring and long-term evaluation of scale-up should be planned before implementation of the updated package of care.

• Health-system changes must be planned and anticipated before implementation, which should enable access to the appropriate next level of expertise.

Involving different stakeholders allowed for consideration of the diverse circumstances across the country and for brainstorming unique solutions tailored to the challenges, for example the development of new strategies to manage the increased patient workload due to better identification of hypertensive disorders of pregnancy. The National Executive Council has since further investigated the management of hypertensive disorders of pregnancy in South Africa and developed new guidelines. The Council has also supported the concept of next-level of expertise – an innovative approach that promotes having some medium-high expert services at the primary health-care level to increase accessibility to higher-level care for all women. The extensive engagement, planning and training for implementation was continually repeated and based on lessons learnt through the country’s scale-up of the Essential Steps in Managing Obstetric Emergencies programme.

Important next steps include impact analyses on maternal and perinatal outcomes; understanding implementation strategies to improve access to services and cost to the health system; and documenting the perspectives of health-care providers and women on the timing, number, content and quality of antenatal care contacts in the context of implementation.

In conclusion, these early results show a promising increase in total antenatal care contacts and hypertension detection. However, implementation of the eight-contact antenatal care model poses new challenges to the health-care system that require prior planning, active monitoring and proactive management. As countries update their antenatal care policies in line with the 2016 WHO antenatal care guideline, our experience and learning from South Africa can help to inform implementation in other countries and the development of an antenatal care monitoring framework.^27^^–^^29^
